# Metabolomics and Inflammatory Mediator Profiling for the Differentiation of Life-Threatening and Non-Severe Appendicitis in the Pediatric Population

**DOI:** 10.3390/metabo11100664

**Published:** 2021-09-28

**Authors:** Nusrat S. Shommu, Jaime Blackwood, Craig N. Jenne, Ari R. Joffe, Dori-Ann Martin, Beata Mickiewicz, Mary Brindle, Robin Eccles, Hans J. Vogel, Graham C. Thompson, on behalf of the Alberta Sepsis Network

**Affiliations:** 1 Bio-NMR Centre, Department of Biological Sciences, University of Calgary, Calgary, AB T2N 1N4, Canada; nsshommu@ucalgary.ca (N.S.S.); vogel@ucalgary.ca (H.J.V.); 2 Department of Pediatrics, Cumming School of Medicine, University of Calgary, Calgary, AB T2N 1N4, Canada; jaime.blackwood@ahs.ca (J.B.); dori-ann.martin@albertahealthservices.ca (D.-A.M.); bmmickie@ucalgary.ca (B.M.); 3 Department of Critical Care Medicine, Cumming School of Medicine, University of Calgary, Calgary, AB T2N 1N4, Canada; cnjenne@ucalgary.ca; 4 Department of Microbiology, Immunology and Infectious Disease, University of Calgary, Calgary, AB T2N 1N4, Canada; 5 Department of Pediatrics, University of Alberta, Edmonton, AB T6G 2R3, Canada; ari.joffe@ahs.ca; 6 Department of Surgery, Cumming School of Medicine, University of Calgary, Calgary, AB T2N 1N4, Canada; mary.brindle@ahs.ca (M.B.); robin.eccles@ahs.ca (R.E.); 7 Department of Community Health Sciences, Cumming School of Medicine, University of Calgary, Calgary, AB T2N 1N4, Canada; 8 Department of Emergency Medicine, Cumming School of Medicine, University of Calgary, Calgary, AB T2N 1N4, Canada

**Keywords:** appendicitis, child, bio-profiling, metabolomics, inflammatory protein mediators

## Abstract

While children with appendicitis often have excellent clinical outcomes, some develop life-threatening complications including sepsis and organ dysfunction requiring pediatric intensive care unit (PICU) support. Our study applied a metabolomics and inflammatory protein mediator (IPM) profiling approach to determine the bio-profiles of children who developed severe appendicitis compared with those that did not. We performed a prospective case-control study of children aged 0–17 years with a diagnosis of appendicitis. Cases had severe disease resulting in PICU admission. Primary controls had moderate appendicitis (perforation without PICU); secondary controls had mild appendicitis (non-perforated). Serum samples were analyzed using Proton Nuclear Magnetic Resonance (^1^H NMR) Spectroscopy and Gas Chromatography-Mass Spectrometry (GC-MS); IPM analysis was performed using plasma bead-based multiplex profiling. Comparisons were made using multivariate data statistical analysis. Fifty-three children were included (15 severe, 38 non-severe). Separation between severe and moderate appendicitis demonstrated excellent sensitivity and specificity (100%, 88%; 14 compounds), separation between severe and mild appendicitis also showed excellent sensitivity and specificity (91%, 90%; 16 compounds). Biomarker patterns derived from metabolomics and IPM profiling are capable of distinguishing children with severe appendicitis from those with less severe disease. These findings provide an important first step towards developing non-invasive diagnostic tools for clinicians in early identification of children who are at a high risk of developing severe appendicitis.

## 1. Introduction

Appendicitis is the most common non-traumatic surgical emergency in children [1]. It has been estimated that appendicitis related health care cost surpasses $680 million per year in the United States [2]. Moreover, appendicitis has the second highest litigation rate in pediatric emergency medicine [3]. Pediatric patients who develop severe disease need substantial critical care resources and prolonged hospital stays in order to manage serious consequences, such as intra-abdominal sepsis, organ dysfunction and, at times, death. Although severe appendicitis can be life-threatening for children, only a limited number of studies are reported in this area. This small body of literature demonstrates a case fatality rate that is significantly higher in children less than 10 years of age compared to all other age groups [4,5]. Factors associated with appendicitis-related morbidity and mortality include sepsis, anesthesia- or surgically-related complications, pre-existing comorbidities, age and male sex [6].

Appendicitis in the pediatric population often has an atypical presentation, particularly in young children who may exhibit several extra-intestinal non-specific symptoms, such as crying, lethargy, and anorexia and a paucity of the characteristic signs [1,7,8]. Moreover, this age group often lacks the developmental capacity to properly describe their symptoms and pain. These challenges result in delayed and/or missed diagnosis, which can increase perforation rate by 50% [1]. While there are many studies related to pediatric appendicitis diagnostic strategies including clinical scoring systems (e.g., Alvarado Score, Pediatric Appendicitis Score) [9,10,11], traditional laboratory tests of infection and inflammation (i.e., white blood cell count, erythrocyte sedimentation rate, C-reactive protein) [12,13,14,15] and imaging studies [16,17,18,19], there are obvious knowledge gaps related to our ability to accurately diagnose appendicitis and predict disease progression. What remains unknown is why some children rapidly progress to perforation and subsequent severe conditions such as sepsis and organ dysfunction while others remain physiologically stable throughout the course of the illness.

A recent focus on bio-profiling through Nuclear Magnetic Resonance (NMR) spectroscopy and/or Mass Spectrometry (MS) [20,21] and cytokine analyses [22,23] has identified biomarker panels that accurately separate specific populations and point to potential target pathways allowing for innovation in therapeutic options. NMR and MS platforms are used to detect various metabolites, the low molecular weight compounds which characterize the intermediate and final products of the metabolic pathways within an organism. The NMR technique is quantitative and highly reproducible [24]; however, NMR has the drawback of low sensitivity. By using NMR in combination with more sensitive methodologies such as MS, it is possible to quantitatively detect a wide range of metabolites. Similarly, using multiplexed cytokine/chemokine analysis, it is possible to simultaneously profile numerous inflammatory protein mediators and obtain a better understanding of the host immune response under differing physiological conditions [22,23]. These analytical approaches have also empowered the comparison of metabolic and protein mediator profiles, thus identifying potential biomarkers for disease diagnosis and prognosis. In addition, requirement of a small sample volume makes this approach quite convenient for disease diagnosis. In previous studies, we have combined metabolomics and protein mediator profiling data for differentiating pediatric appendicitis patients from pediatric controls (patients with no abdominal pain), pediatric appendicitis patients from non-appendicitis patients presenting with similar abdominal pain, and children with simple appendicitis from those with perforated appendicitis [25,26,27]. In the present study, we have applied a similar integrated profiling approach for identifying differences in the bio-profiles of children with appendicitis who require critical care management compared to those who do not. We anticipate that this approach will (1) allow clinicians to accurately predict disease severity and provide early escalation of care to those at risk of severe disease, and (2) provide a better understanding of the underlying pathophysiology.

## 2. Results

### 2.1. Sample Description

This study evaluated samples from 53 pediatric patients (15 ‘severe appendicitis’, 9 ‘moderate appendicitis’ and 29 ‘mild appendicitis’). Table 1 provides demographic and clinical characteristics of the patient cohorts. A total of 86 known metabolites by ^1^H NMR and GC-MS, and 57 protein mediators by Luminex techniques could be consistently detected and analyzed for each study subject.

### 2.2. Metabolic Profile

**Figure 1:**
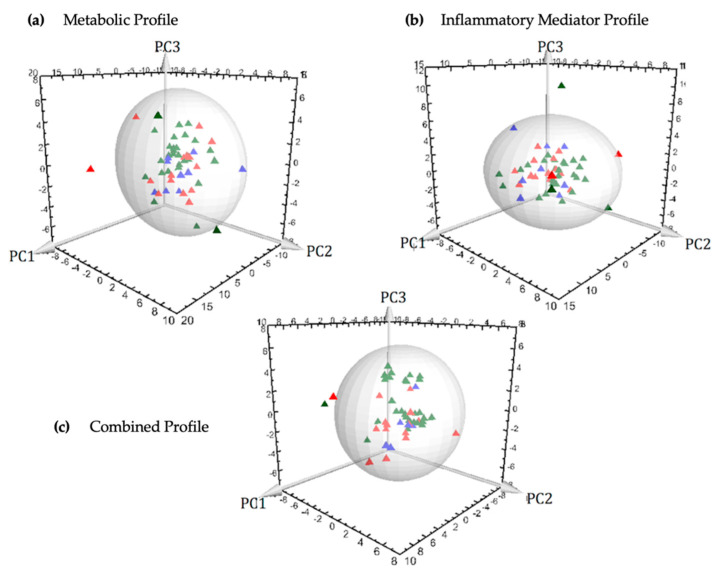
Three-dimensional PCA-X score scatter plots of pediatric patients with appendicitis using (**a**) metabolic profile, (**b**) inflammatory mediator profile, (**c**) combined metabolic and inflammatory mediator profile. Each plot is based on three principal components - PC1, PC2 and PC3. Red diamonds represent distribution of pediatric patients with severe appendicitis, blue diamonds represent patients with moderate appendicitis and green diamonds represent patients with mild appendicitis.

**Figure 2:**
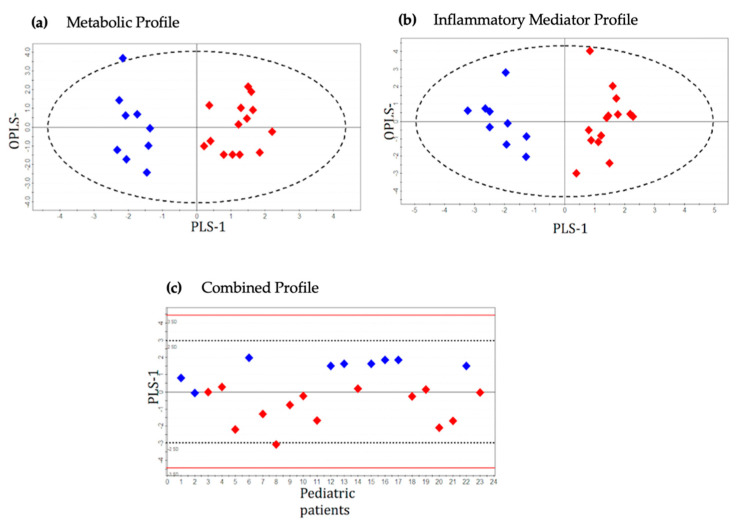
OPLS-DA score scatter plots distinguishing pediatric patients with severe appendicitis and moderate appendicitis using (**a**) metabolic profile (R^2^Y = 0.90, Q^2^ = 0.70), (**b**) inflammatory mediator profile (R^2^Y = 0.66, Q^2^ = 0.56) and (**c**) combined metabolic and inflammatory mediator profile (R^2^Y = 0.78, Q^2^ = 0.65). Red diamonds represent distribution of pediatric patients with severe appendicitis, and blue diamonds represent patients with moderate appendicitis. OPLS—Orthogonal Partial Least Squares; PLS—Partial Least Squares.

**Figure 3:**
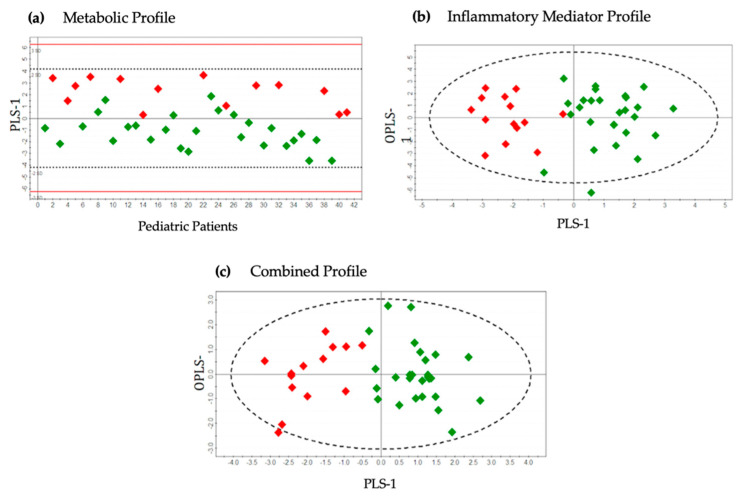
OPLS-DA score scatter plots distinguishing pediatric patients with severe and mild appendicitis using (**a**) metabolic profile (R^2^Y = 0.60, Q^2^ = 0.48), (**b**) inflammatory mediator profile (R^2^Y = 0.63, Q^2^ = 0.55), and (**c**) combined metabolic and inflammatory mediator profile (R^2^Y = 0.65, Q^2^ = 0.57). Red diamonds represent distribution of pediatric patients with severe appendicitis, and green diamonds represent patients with mild appendicitis. OPLS—Orthogonal Partial Least Squares; PLS—Partial Least Squares.

The common metabolites that were detected from ^1^H NMR and GC-MS platforms illustrated similar trends in different patient groups. Three principal components (PC1, PC2 and PC3) were calculated to build the unsupervised PCA model for the metabolic dataset (^1^H NMR and GC-MS) contributing 16.9%, 10.1% and 6.6% of variation, respectively (Figure 1a). Three outliers (two mild and one severe appendicitis) were identified and excluded from further downstream analysis. Supervised OPLS-DA models consisted of only two classes. The severe appendicitis group was separately compared with moderate and mild appendicitis groups (Figure 2a and Figure 3a). The score scatterplots for each of the OPLS-DA analyses illustrate substantial separation of the two groups with strong variance and predictive ability (Figure 2a and Figure 3a, Table 2). The R^2^Y:Q^2^ ratio for the model comparing severe and moderate groups (0.90:0.70) is higher compared to severe vs. mild groups (0.60:0.48); both models show excellent significance in statistical parameters including sensitivity and specificity (severe vs. moderate—1.00:0.86, severe vs. mild—0.71:0.89, respectively), CV-ANOVA *p*-values, and AUROC values (Table 2).

### 2.3. Inflammatory Protein Mediator Profile

The unsupervised PCA model for the inflammatory protein mediator dataset was built based on three principal components (PC1, PC2 and PC3), contributing to 18.8%, 13.1% and 10.2% of the variation, respectively (Figure 1b). Two outliers were identified (one mild and one severe appendicitis) and excluded from subsequent analysis. Supervised OPLS-DA methodology was applied to compare the variances between each pair of case-control groups: severe vs. moderate (Figure 2b) and severe vs. mild (Figure 3b). Score scatterplots for the OPLS-DA analysis demonstrate separation of each of the two classes having considerably strong variance and predictive values (severe vs. moderate: R^2^Y = 0.66, Q^2^ = 0.56; severe vs. mild: R^2^Y = 0.63, Q^2^ = 0.55, Figure 2b and Figure 3b and Table 2). The strength of the models is supported by the sensitivity and specificity (severe vs. moderate—0.92:0.80, severe vs. mild—0.92:0.90, respectively), AUROC and CV-ANOVA *p*-values (Table 2).

### 2.4. Integrated Metabolic and Protein Mediator Profile

**Figure 4:**
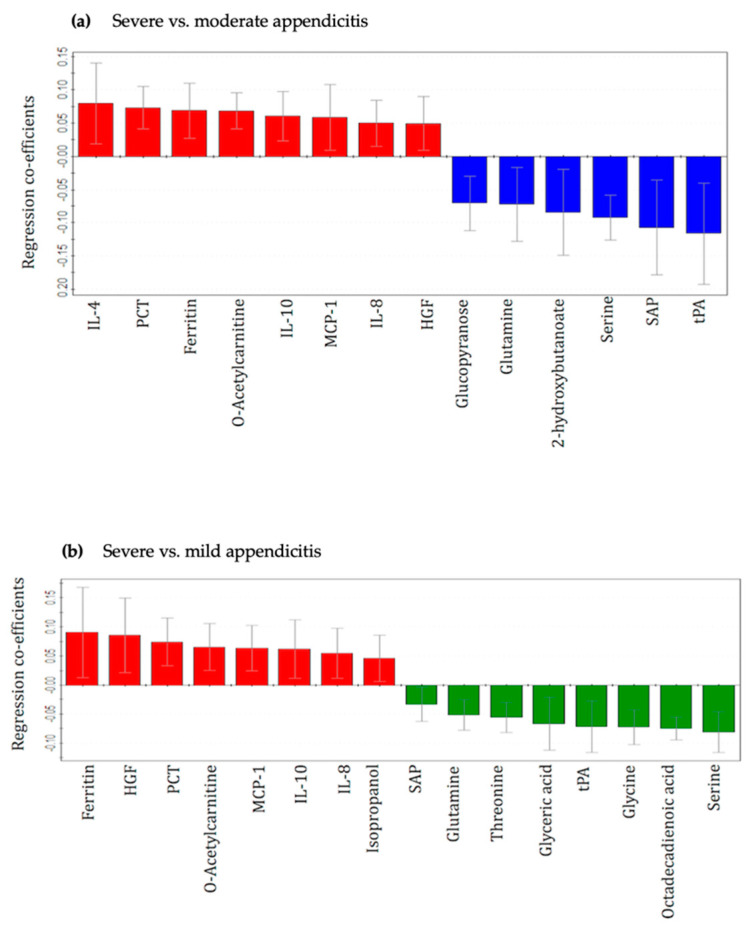
Regression coefficient plots for the statistically significant (*p* < 0.05) metabolites and inflammatory mediators distinguishing children with (**a**) severe vs. moderate appendicitis and (**b**) severe vs. mild appendicitis. Red bars with positive coefficient values represent increased concentrations in the severe appendicitis children, blue bars and green bars with negative values represent reduced concentration in the severe appendicitis patients, compared with moderate and mild appendicitis. IL—Interleukin, HGF—Hepatocyte growth factor, PCT—procalcitonin, MIF—macrophage migration inhibitory factor, MCP-1—Monocyte chemoattractant protein-1, SAP—Signaling lymphocyte activation molecule (SLAM)-associated protein and tPA—tissue plasminogen activator.

A total of 16 metabolites and 15 inflammatory protein mediators provided the most significant contribution for the separation between severe and control patient groups in the corresponding OPLS-DA models. These 31 compounds were combined to develop an integrated metabolic and inflammatory protein mediator dataset. The unsupervised PCA model for the integrated dataset was based on three principal components: PC1 = 24.6%, PC2 = 13.7% and PC3 = 9.6% (Figure 1c). Two outliers from the ‘severe’ group were identified and excluded from the supervised analysis. Supervised OPLS-DA models were built to illustrate the differences between case and control patient classes. Score scatterplots demonstrated excellent separation of each of the two pairs of groups with strong R^2^Y and Q^2^ values (severe vs. moderate—0.75:0.65; severe vs. mild—0.65:0.57, Figure 2c and Figure 3c and Table 2). The combined dataset analysis also revealed high sensitivity and specificity (severe vs. moderate—1.00:0.88, severe vs. mild—0.91:0.90, respectively), and AUROC values for the models (Table 2). A bio-pattern comprised 5 serum metabolites and 9 inflammatory protein mediators that contributed significantly (*p* < 0.05) for the separation between pediatric patients with severe and moderate appendicitis (Figure 4a). Similarly, a bio-pattern constituting of 7 significant metabolites and 9 inflammatory protein mediators was detected for the distinction between the severe and mild appendicitis groups (Figure 4b).

To confirm that the exclusion of outliers from the supervised analyses did not bias our results, we re-developed the OPLS-DA models including all outliers, for all three datasets. The exclusion had negligible influence on the discriminative and predictive ability of the models (Table 3).

## 3. Discussion

This study has provided us with a novel opportunity to explore biochemical processes underlying the most severe presentations of appendicitis. We believe our study is unique in its use of an expansive, multi-modal biomarker profiling approach. Analyses were performed through advanced quantitative techniques capable of analyzing hundreds of different metabolites and inflammatory protein mediators with high sensitivity. From this arsenal of biological compounds, we identified biomarker fingerprints that distinguished severe appendicitis from mild and moderate appendicitis for the children included in our study. Our analyses demonstrated promising results separating the cohorts of different severity with strong predictive values, sensitivity, specificity and AUROC. The excellent performance of our models suggests that the identified biomarker patterns distinguishing severe appendicitis from moderate and mild presentations of the disease may have clinical significance.

Several significant metabolites in the bio-profiles were identified as key in separating the children with severe appendicitis from those with mild and moderate appendicitis. This finding implies that significant disruption of multiple metabolic processes underlies the development of severe disease pediatric patients with appendicitis. The depleted level of 2-hydroxybutanoate and increased o-acetylcarnitine indicate higher fatty acid degradation during severe appendicitis [28,29,30,31] compared to the moderate condition (Figure 4). Similarly, comparison with the mild disease group revealed increased levels of isopropanol and o-acetylcarnitine along with reduced levels of octadecadienoic acid in the severe group indicating elevated fatty acid degradation. Our findings suggest that fatty acid breakdown is induced during appendicitis, which elevates with the severity of the disease. However, whether this fatty acid manifestation is the cause or effect of appendicitis and its severity, is not clear. Another pathway, glycine, serine and threonine metabolism, is markedly disrupted during severe appendicitis as implied by the presence of a number of metabolites from this pathway in the detected biopatterns. For instance, the metabolites threonine, glyceric acid, glycine and serine detected in the bio-profile of severe vs. mild, and the metabolite serine found in severe vs. moderate analysis are essential parts of the glycine, serine and threonine metabolism pathway [28,29,30,31]. Depletion of glutamine in both bio-profiles indicates that one or more of the pathways, such as arginine biosynthesis, purine metabolism, pyrimidine metabolism, and alanine, aspartate and glutamate metabolism may be affected because glutamine is an essential component of all these pathways [28].

Appendicitis is characterized by an excessive inflammatory response [32], which is also reflected by the identification of a notable number of inflammatory mediators identified in our biomarker patterns. The levels of inflammatory markers procalcitonin, ferritin, interleukin-10 (IL-10), monocyte chemoattractant protein-1 (MCP-1), IL-8 and hepatocyte growth factor (HGF) were higher whereas the concentrations of signaling lymphocyte activation molecule (SLAM)-associated protein (SAP) and tissue plasminogen activator (tPA) were reduced in the children with severe appendicitis compared to children with moderate and mild appendicitis (Figure 4).

Many of these inflammatory biomarkers are consistent with previous studies. For instance, as an essential strategy of the host immune response, ferritin is almost always elevated during infection to chelate iron and deprives the invading pathogen from this essential nutrient [33,34]. We have also identified elevated ferritin in our previous studies that compared appendicitis with non-appendicitis, and perforated with simple appendicitis, which suggests that with increasing severity of appendicitis inflammation, the concentration of ferritin also increases in the host blood. We have identified elevated levels of IL-10 in the severe group compared to both control groups. In addition to be an important inflammatory biomarker for acute appendicitis [35,36], IL-10 has also been found in other studies to be increased during severe appendicitis [37,38]. The concentration of IL-8 has been elevated during acute appendicitis [32,39]; detection of this interleukin in our study implies that it’s level also increases with the increasing complexity of the disease. Similar to our finding, previous studies reported that procalcitonin is an indicator of the severity of acute appendicitis [4]. However, in contrast to our finding, the cytokine IL-4 has been found to be elevated in mild appendicitis when compared with the more severe form [38].

Overall, the metabolites and protein mediators detected from pediatric patients provide distinctive biomarker patterns to distinguish severe and non-severe appendicitis. Although various studies in the literature focused on selected inflammatory biomarkers of appendicitis, gaps in timely and accurate diagnosis and prediction of disease progression continue to exist. There is very little literature that reports on why some children develop life-threatening appendicitis, and there are still knowledge gaps about why some children with perforated appendicitis occasionally develop serious complications while most of the others respond well to ED and surgical management. Our study is an important initial step towards answering this question.

The presence of a small number of compounds in the detected bio-patterns should make it possible to develop a time-sensitive point of care aid for the emergency pediatric physicians to identify children with risk of developing severe appendicitis. A similar type of biomarker phenotyping approach using integrated metabolomics and protein mediator profiling has shown promising outcomes in early diagnosis and prognosis of sepsis [40,41]. Hence, it is rational to suggest that the promising findings from our study have also the potential to improve the prediction of disease progression in children with appendicitis.

Our results demonstrated that the separation between severe and moderate conditions were somewhat better than that between severe and mild conditions. It is possible that children with mild appendicitis have yet to establish a common set of metabolic derangements that converge on a common profile which would be identified as different from severe disease. In addition, the differences in sample sizes of the cohorts might explain this outcome [42,43]. In an attempt to minimize statistical bias [42,43], we maintained a ratio close to 1:2 during our analyses. Another limitation is that while blood samples were collected from the majority of the children before antibiotic treatment, some children had their samples drawn in PICU (albeit at the earliest time point achievable) due to severity of the condition. These children would have undergone additional resuscitation efforts including antibiotics, fluids, inotropes etc. Finally, due to the small sample size of this investigation, follow-up studies will be needed to confirm our findings across ages.

## 4. Materials and Methods

### 4.1. Study Design, Setting and Population

We performed a post hoc sub-group analysis of children aged 0–17 years who had been prospectively enrolled in the Alberta Sepsis Network (ASN) study and who had pathologically proven appendicitis. The ASN study was investigating the metabolic and inflammatory processes in children managed in the Emergency Department (ED) or the Pediatric Intensive Care Unit (PICU) for an infectious illness [44]. Children were eligible for enrolment into the ASN study if they met criteria for systemic inflammatory response syndrome, had a blood culture performed and antibiotics ordered. Those not expected to survive ≥24 h, refusing intubation or vasoactive infusions (e.g., palliative care), or already having had severe sepsis for ≥48 h (defined as sepsis with cardiovascular dysfunction, acute respiratory distress syndrome, or two other organ dysfunctions) were excluded. Enrolment occurred at the two pediatric tertiary hospitals in the province of Alberta, Canada.

In this current analysis, cases (*severe cohort*) were defined as children requiring PICU admission due to the severity of appendicitis, following consultation between the most responsible clinician (Emergency, Surgery) and the PICU attending clinician. Factors associated with a decision for PICU admission include, but are not limited to: requirement for significant fluid resuscitation, requirement of vasoactive support, initiation of mechanical ventilation (including both invasive and non-invasive). The primary control group (*moderate cohort*) included those with appendiceal perforation but without PICU admission. The secondary control group (*mild cohort*) included children with positive pathology for appendicitis but neither appendiceal perforation nor PICU admission. The diagnosis of appendicitis was defined by either (a) pathology report indicating any evidence of acute inflammation of the appendix, or (b) surgical placement of a percutaneous drain (for abscess collection related to the appendix) with interval appendectomy. Perforation of the appendix was defined by (a) pathology report indicating any level of perforation/rupture/disruption of the wall of the appendix, or (b) placement of a percutaneous drain for an abscess collection related to the appendix.

Approval for this study was obtained from the Health Research Ethics Board of the University of Alberta and the Conjoint Health Research Ethics Board of the University of Calgary. Children with severe life-threatening disease requiring active resuscitation and PICU admission were enrolled using a deferred consent model; for children with moderate and mild disease, informed consent/assent to participate in the study was obtained from the child and/or their parent/caregiver.

### 4.2. Sample Collection

Blood samples were obtained from children at the earliest possible time following enrolment and within 24 h after admission, whether that was in the PICU or ED. The volume of blood obtained was as follows: 2 mL from infants (0–<1 years), 4 mL from preschool and school-aged (1–12 years), and 8 mL from teenagers (13–17 years). For metabolic profiling, whole blood was collected into serum (red top) tubes. Immediately after collection, blood samples were gently inverted several times and allowed to clot at room temperature for an hour. The samples were then placed on ice for 2 h. The samples were centrifuged at 1200× *g* for 10 min at 4 °C and serum was then collected in a 4 mL cryovial and stored at −70 °C until distribution for metabolomics analysis. For inflammatory mediator profiling, whole blood was collected into a sodium-heparin plasma tube. The tubes were then gently inverted several times, and immediately placed on ice. The samples were centrifuged at 1200× *g* for 10 min at 4 °C in a swinging bucket centrifuge and the separated plasma was collected in a 4 mL cryovial. The plasma samples were stored at −70 °C until distribution for multiplex analysis.

### 4.3. Proton Nuclear Magnetic Resonance (^1^H NMR) Spectroscopy

Samples were prepared for the acquisition of ^1^H NMR spectra following a procedure described in earlier studies [44,45]. Briefly, serum samples were filtered using 3 kDa NanoSep microcentrifuge filters to remove large molecules. Sodium azide and D_2_O were added to the filtrate and the final pH of each sample was adjusted to 7.00 ± 0.04 by using phosphate buffer. The prepared samples were analyzed on a 600 MHz Bruker Ultrashield Plus NMR spectrometer (Bruker BioSpin Ltd., Milton, ON, Canada) to obtain ^1^H NMR spectra using the standard Bruker 1D spectroscopy pulse program ‘noesypr1d’ [46,47]. Each spectrum was manually processed for phasing, baseline correction, and referencing to the DSS peak at 0.0 ppm using the Chenomx NMR Suite 7.5 software (Chenomx Inc., Edmonton, Canada). The same software was used for profiling of each sample spectrum, which implicates identification of different metabolites by their distinctive spectral signature using an external metabolite reference database [46,47].

### 4.4. Gas Chromatography-Mass Spectrometry (GC-MS)

A previously described protocol was followed for sample extraction and derivatization for GC-MS^21^. In brief, metabolites from serum were extracted using a two-phase chloroform and methanol mixture. The aqueous layer was then separated and vacuum-dried by SpeedVac (Eppendorf, Germany). The metabolites were subsequently derivatized by methoxyamine hydrochloride in pyridine solution and N-methyl-N-(trimethylsilyl) trifluoroacetamide. An internal standard (phenylalanine D5) was used for acquiring the relative concentrations of the analytes. Mass spectra were obtained using Agilent chromatograph 7890A (Agilent Technologies Canada, Inc, Mississauga, ON, Canada) coupled with a Time-of-Flight Mass Spectrometer (Waters Corp., Milford, MA, USA); the MS range used for scanning was 50–800 *m*/*z.* The generated GC-MS spectra were then processed using the Metabolite Detector software (version 2.06, Technische Universität Carolo-Wilhelmina zu Braunschweig, Braunschweig, Germany) followed by identification of metabolites based on the GOLM metabolome database [48].

### 4.5. Inflammatory Mediator Profiling

The inflammatory mediators in the plasma samples were analyzed using three human cytokine and chemokine assay kits (Bio-Plex Pro Human Cytokine 21-Plex Assay, Bio-Plex Pro Human Cytokine 27-Plex Assay and Bio-Plex Pro Human Acute Phase Protein 5- + 4- Plex Assay) manufactured by Bio-Rad Laboratories, Inc. (Hercules, CA, USA). A Luminex 200 apparatus (Applied Cytometry Systems, Sheffield, UK) was used for reading the assay plates by following the manufacturer guidelines. Detection and analysis of the inflammatory protein mediators were done using the software Bio-Plex Manager 6.0 (Bio-Rad Laboratories, Inc.). Samples were run in duplicate and the data for a replicate were considered as a missing value if two replicates had a coefficient of variance greater than 20%.

### 4.6. Statistical Analysis

The SIMCA-P+ software (v12.0.1; Umetrics, Umeå, Sweden) was used for performing multivariate data statistical analysis of the metabolite and protein mediator profiles [42,43,49,50,51,52]. The common metabolites that were detected by both ^1^H NMR and GC-MS techniques were averaged and all the metabolites identified from the two platforms were combined to generate the metabolomics profile. The metabolomics and protein mediator datasets were first analyzed separately, then combined and re-analyzed. Metabolites and mediators having >20% missing values were excluded from the statistical analysis followed by data preprocessing (median fold change normalization, logarithmic transformation, centering, and unit variance scaling), for separate and combined datasets [52].

An initial overview of the multivariate datasets was obtained by unsupervised principal component analysis (PCA), which also identified outliers situated beyond of the 95% confidence interval [42]. The outliers were excluded from further analysis to prevent bias in the supervised models. Supervised orthogonal partial least squares discriminant analysis (OPLS-DA) was performed. Potentially relevant metabolites were selected using the variable importance to projection (VIP); those variables with VIP >1 were included in developing the OPLS-DA models. R^2^Y (the percentage of variation explained by the model), Q^2^ (the predictive ability of the model) and CV-ANOVA (Cross-Validated Analysis of Variance) *p*-value were calculated for the OPLS-DA models using a sevenfold cross-validation method. Regression coefficients for the OPLS-DA models were calculated from the combined dataset to detect the most critical compounds contributing to the class separations [51]. Those metabolites and inflammatory mediators which demonstrated significant differences in concentration (*p* < 0.05) between the classes were considered for biomarker fingerprint development. Clinically important parameters including sensitivity (Sn), specificity (Sp), and area under the receiver operating characteristic curve (AUROC) [53] were calculated for each OPLS-DA model from sample class prediction during sevenfold cross-validation (YpredCV) in the SIMCA-P+ software. Metz ROC (The University of Chicago, IL, USA) was used to calculate the AUROC values. MetaboAnalyst 3.0 was used to identify the metabolic pathways perturbed during severe appendicitis [28].

## 5. Conclusions

We have explored the metabolic and inflammatory processes underlying life-threatening presentations of appendicitis in children on a provincial level and obtained bio-profiles that separate the children with severe appendicitis from those with mild and moderate presentations of the disease. We have also identified key metabolic pathways that are disrupted during severe appendicitis. The results from this project have the potential to improve the diagnosis, management and outcomes of children presenting with appendicitis, in particular those with life-threatening illness. In addition, identifying pathways responsible for the progression of disease holds the potential for future innovation in therapeutics aimed at mitigating systemic inflammatory response accompanying severe disease, thus leading to improved patient outcomes. The promising pilot data from this study have been used by our team to inform a large multi-center follow-up study focused on validating the bio-profiles developed by metabolomics and protein mediator analyses. Additional future studies could ultimately result in the development of a highly-accurate, accessible, cost-effective, rapid (<1 h), minimally invasive (capillary finger-prick) point-of-care diagnostic test.

## Figures and Tables

**Table 1:** Demographics of children presenting with severe, moderate and mild appendicitis.

Patient Demographic	Severe Appendicitis	Moderate Appendicitis	Mild Appendicitis
	(*n* = 15)	(*n* = 9)	(*n* = 29)
Age, median (IQR)	10 (6–14)	9 (8–14)	11 (9–13)
Sex—Female; *n* (%)	6 (40.0)	6 (40.0)	13 (44.8)
Documented RLQ Pain, *n* (%)	12 (80.0)	7 (77.8)	23 (79.3)
Documented Nausea, *n* (%)	6 (40.0)	5 (55.6)	19 (65.5)
Documented Vomiting, *n* (%)	10 (66.7)	5 (55.6)	19 (65.5)
Documented Anorexia, *n* (%)	12 (80.0)	6 (66.7)	17 (58.6)
Documented Fever in the ED, *n* (%)	11 (73.3)	5 (55.6)	16 (55.2)
WBC in the ED; mean (SD)	15.9 (2.1)	19.8 (1.5)	17.1 (1.2)
Neutrophils in the ED, mean (SD)	13.0 (2.0)	17.0 (1.5)	14.2 (1.1)
US completed in the ED, *n* (%)	10 (66.7)	8 (88.9)	29 (100.0)
CT completed in the ED, *n* (%)	2 (13.3)	1 (11.1)	1 (3.5)
ED Disposition, *n* (%)			
Surgery Ward	0 (0.0)	0 (0.0)	5 (17.2)
OR	10 (66.7)	9 (100.0)	24 (82.8)
PICU	5 (33.3)	0 (0.0)	0 (0.0)
PRISM 3, median (IQR)	3 (0–4)	-	-
PELOD (Day 1), median (IQR)	2 (1–11)	-	-
Ventilator Days, median (IQR)	0 (0–2)	-	-
Inotrope Days, median (IQR)	1 (0–1)	-	-
PICU Length of Stay (days), median (IQR)	3 (3–4)	-	-

CT—Computed Tomography; ED—Emergency Department; IQR—Interquartile Range; OR—Operating Room; PELOD—Pediatric Logistic Organ Dysfunction Score; PICU—Pediatric Intensive Care Unit; PRISM—Pediatric Risk of Mortality Score; RLQ—Right Lower Quadrant; SD—standard deviation; US—Ultrasound; WBC—White Blood Cell.

**Table 2:** Summary statistics from OPLS-DA models differentiating each pair of pediatric patient groups.

Analysis		Sn:Sp	AUROC	CV-ANOVA *p*-Value	R^2^Y:Q^2^
**Severe vs. moderate** **appendicitis**	Metabolic profile	1.00:0.86	1.00 ± 0.00	1.3 × 10^−4^	0.90:0.70
	Inflammatory mediator profile	0.92:0.80	0.92 ± 0.08	9.9 × 10^−4^	0.66:0.56
	Combined profile	1.00:0.88	1.00 ± 0.00	2.4 × 10^−4^	0.78:0.65
**Severe vs. mild** **appendicitis**	Metabolic profile	0.71:0.89	0.93 ± 0.04	4.8 × 10^−6^	0.60:0.48
	Inflammatory mediator profile	0.92:0.90	0.96 ± 0.03	9.3 × 10^−6^	0.63:0.55
	Combined profile	0.91:0.90	0.97 ± 0.03	5.1 × 10^−6^	0.65:0.57

Sn—Sensitivity, Sp—Specificity, AUROC—Area Under the Receiver Operating Characteristic curve, CV-ANOVA—Cross Validated Analysis of Variance, SD—Standard Deviation.

**Table 3:** Comparison of R2Y:Q2 values calculated for the OPLS-DA models with and without outliers.

Analysis		R2Y:Q2 Outliers Excluded	R2Y:Q2 Outliers included
	Metabolic profile	0.90:0.70	0.83:0.70
**Severe vs. moderate appendicitis**	Inflammatory mediator profile	0.66:0.56	0.61:0.53
	Combined profile	0.78:0.65	0.73:0.59
**Severe vs. mild appendicitis**	Metabolic profile Inflammatory mediator profile Combined profile	0.60:0.48	0.63:0.40
		0.63:0.55	0.62:0.55
		0.65:0.57	0.63:0.56

## Data Availability

The data presented in this study are available on request from the corresponding author. Data is not publicly available because we do not have ethics approval to publish our data set.
